# On the Changes of Form of Mammalian Blood-Corpuscles

**Published:** 1864-06

**Authors:** 


On the Changes of Form of Mammalian Blood-corpuscles.—The *Centralblatt für die Medicinischen Wissenchaften* of the 5th ult., contains an important paper by Dr. Klebs, assistant at the Pathological Institute at Berlin, on "The Changes of Form of the Red Blood-corpuscles in Mammalia." Dr. Klebs finds that, when the disk-shaped blood-corpuscles, which are joined together by so-called *rouleaux*, in the cooled blood of dead animals, are heated to the temperature of the body, care being taken to prevent evaporation, the following change of shape takes place. The substance of the corpuscle begins to accumulate at one part of the margin, and the outline of the corresponding opposite part becomes flattened and afterwards indented. The substance then generally accumulates more strongly at the ends caused by the indentation; hard protuberances (*zacken*, Dr. Klebs calls them), are formed, which project over the margin, and a broader protuberance is often formed first, and then divides into two smaller ones. This process is repeated over the whole of the disc of the blood corpuscle, which thus becomes quadrangular, or, more frequently, pentagonal or hexagonal in shape. A strong affluence towards a point always precedes the formation of the protuberances, which alter their shape very slowly, but constantly; they are seen to be absorbed completely, and new ones then appear near the places of the old ones. The corpuscles with protuberances rest upon the lengthening and shortening ends of these protuberances, and the formation of the latter occasions a very peculiar motion of the corpuscles, inducing a state of constant oscillation, while the liquid is perfectly motionless. When two corpuscles are touching one another with their horizontal protuberances, and vibrating towards each other, slight rotary movements often occur. The protuberances are triangular when in a fresh state and viewed sideways; they do not protrude much above the surface. In the *rouleaux* the terminal blood corpuscles first exhibit the protuberances, and that on the free side. Six various sorts of blood were examined, and all exhibited the phenomenon in the same way; but there were some differences as regards the period when the contraction occurred. The contractibility of the blood-corpuscles survives the death of the individual a considerable time; in the case of some, *rouleaux* are commonly found which separate into forms with protuberances on the application of heat twenty-four hours after death; after forty hours they generally appeared globular, and no longer adhered to one another. Nasse and Bothin state that the corpuscles become ultimately round when immersed in a concentrated solution of salt; but Dr. Klebs finds the shape depends upon what it was previous to immersion, and that the corpuscles with protuberance retain that shape after immersion, at least for a time (allowance being made for the effect of exosmosis), although they appear to be petrified, as it were, and no further movement of contraction is observed. Dr. Klebs has made use of this power of the salt solution to arrest any change of form in the corpuscles to determine what shape these bodies have when circulating in the living animal. A vein of a rabbit was cut open and the wound filled with the salt solution. The result was that the corpuscles, on examination, appeared to be exceedingly thin discs with a great tendency to the formation of folds. The central depression was much more extended than when seen under ordinary circumstances, and the broad margin thinner. A great number of the corpuscles showed the form with protuberances described above. Dr. Klebs, therefore, concludes that the contraction must occur within the blood-vessels. He finishes his paper by giving as his reason for publishing these results of his researches with the least possible delay, and not waiting till he could work up the question from various points of view, that he hopes to secure very numerous fellow-laborers in this most interesting field of research.—*The London Reader.*

